# Assessment of five typical environmental endocrine disruptors and thyroid cancer risk: a meta-analysis

**DOI:** 10.3389/fendo.2023.1283087

**Published:** 2023-10-30

**Authors:** Yuyao Yang, Xiaoyue Bai, Juan Lu, Ronghao Zou, Rui Ding, Xiaohui Hua

**Affiliations:** Department of Occupational Health and Environmental Health, School of Public Health, Anhui Medical University, Hefei, Anhui, China

**Keywords:** environmental endocrine disruptors, thyroid cancer, meta-analysis, PCBs, PBDEs, PAEs, BPA, heavy metals

## Abstract

**Introduction:**

There are conflicting reports on the association between environmental endocrine disruptors (EEDs) and thyroid cancer. This meta-analysis aimed to elucidate the relationship between EEDs and thyroid cancer.

**Methods:**

We searched for epidemiological studies on EEDs and thyroid cancer published in PubMed and Web of Science up to December 2022. We then screened the articles that could extract data on EEDs concentration levels in both thyroid cancer patients and healthy controls. We excluded articles that could not calculate effect sizes, focused on other thyroid diseases, or lacked controls. Standardized mean difference (SMD) was calculated to analyze the association between EEDs and thyroid cancer. We measured the heterogeneity among the included studies using I2, assessed publication bias by Egger’s and Begg’s test, and evaluated article quality using the Newcastle-Ottawa Quality Score (NOS). In the end, fifteen eligible case-control studies were included.

**Results:**

Our comprehensive analysis revealed that polychlorinated biphenyls (PCBs) were negatively associated with thyroid cancer{ SMD = -0.03, 95% confidence interval (CI) = (-0.05, -0.00), P = 0.03}, while polybrominated diphenyl ethers (PBDEs), phthalates (PAEs), and heavy metals were positively associated with thyroid cancer{PBDEs: SMD = 0.14, 95%CI = (0.04, 0.23), P = 0.007; PAEs: SMD = 0.30, 95%CI = (0.02, 0.58), P = 0.04; heavy metals: SMD = 0.21, 95%CI = (0.11, 0.32), P < 0.001}. We did not find a statistically significant relationship between bisphenol A (BPA) and thyroid cancer. Most of the included studies did not show publication bias, except for those on PCBs.

**Discussion:**

Our results indicate that exposure to certain EEDs, such as PBDEs, PAEs, and heavy metals, increases the risk of thyroid cancer. However, further large-scale epidemiological studies and mechanism studies are needed to verify these potential relationships and understand the underlying biological mechanisms.

## 1 Introduction

Thyroid cancer is a common endocrine malignancy, and the incidence of thyroid cancer has increased significantly in recent decades (1). In 2020, the incidence of thyroid cancer ranks 9th in the global cancer incidence spectrum, with the global incidence of thyroid cancer being 10.1/100,000 women, 3.1/100,000 men, and 586,200 new cases (2). From 2005 to 2016, the incidence and mortality of thyroid cancer in China showed an increasing trend over time (3). Thyroid cancer seriously affects people’s life and health, and brings a heavy disease burden. Multiple factors may be associated with an increased incidence of thyroid cancer, including excessive iodine intake, overdiagnosis, exposure to ionizing radiation, environmental endocrine disruptors, thyroid disease history or family history. Currently, ionizing radiation is a confirmed risk factor for thyroid cancer, while the association between other factors and thyroid cancer remains controversial (3–7).

Environmental endocrine-disruptors (EEDs) are defined as exogenous chemicals or mixtures of chemical substances that disrupt any aspect of the action of hormones (7). Humans are exposed to EEDs in various ways in their daily lives. Since the International Endocrine Society issued its first scientific statement in 2009, more people have been paying attention to how EEDs affect human health, and numerous studies have been conducted on the effects and mechanisms of EEDs on thyroid function (8). Extensive experimental studies *in vivo* and *in vitro* have demonstrated that EEDs interfere with thyroid function through a variety of mechanisms (9–12). Furthermore, in populations, several case-control and cohort studies have provided reliable evidence to investigate the potential correlation between exposure to specific chemicals and thyroid cancer (13–16).

To date, there have been many studies on the association between EEDs and thyroid cancer, resulting in conflicting findings. In this meta-analysis, we pooled epidemiological studies on EEDs and thyroid cancer, comparing the concentrations of EEDs in patients with thyroid cancer to those in healthy controls, to evaluate the potential relationship between EEDs exposure and thyroid cancer.

## 2 Materials and methods

### 2.1 Search strategy

A comprehensive search of PubMed and Web of Science databases was conducted for articles published up to December 2022 on the effects of environmental endocrine disruptors on thyroid cancer. The following keywords were used in the search: “Thyroid carcinoma” or “Thyroid Neoplasm” or “Thyriod Carcinoma” or “Thyroid Carcinomas” or “Cancer of Thyroid” or “Thyroid Cancers” or “Thyroid Cancer” or “Thyroid Adenoma” or “Thyroid adenoma” and “Endocrine Disrupting Chemicals” or “Endocrine disruptors” or “Endocrine Disruptor” or “Endocrine Disrupting Chemical” or “Endocrine Disruptor Effect” or “Endocrine Disruptor Effects” or “Bisphenol A” or “Polychlorinated biphenyls” or “phthalates” or “Polybrominated diphenyl ethers” or “heavy metals” or “lead” or “copper” or “arsenic” or “chromium” or “cadmium” or “mercury” or “PAE”. The study included only articles written in English.

### 2.2 Study selection

Two researchers (YYY and XYB) independently conducted the identification of potentially eligible studies and data extraction of related studies, and assessed the quality of the studies included in the article. All differences were resolved by discussion with the third-party inspector (XHH).

Studies with the following characteristics were considered to meet the inclusion criteria: (1) studies focusing on the relationship between EEDs and thyroid cancer; (2) observational epidemiological studies (i.e., cohort, cross-sectional, or case-control studies); (3) the levels of EEDs exposure in humans is determined in biological samples (plasma, serum, or urine); (4) the study population was thyroid cancer patients and healthy controls; (5) to provide data on levels of EEDs in thyroid cancer patients and healthy controls; (6) no other diseases, no drugs that may affect serum or urinary endocrine disruptors levels.

Studies with one or more of the following characteristics were excluded from this meta-analysis: (1) reviews, editorials, letters, case reports or non-human studies; (2) the serum or urine levels of EEDs were not provided in both thyroid cancer patients and healthy controls; (3) results could not be extracted or converted to standardized mean difference (SMD) and 95%CI.

### 2.3 Data extraction

Data extraction was independently performed by two researchers (YYY and XYB) using the standardized data extraction tables. The detailed data extraction tables included the following items: first author, year of publication, country, period of study, study design, sample size, measurement methods, and NOS scores.

### 2.4 Quality assessment

Two researchers (YYY and XYB) independently assessed the quality of eligible studies using the Newcastle-Ottawa Quality Scale (NOS) (17). The scale assesses research quality through eight questions from three dimensions of study population selection, comparability, exposure assessment or outcome assessment, with a full score of 9. Studies with an overall score of 7-9 are considered to be of high quality and included in this meta-analysis. All disagreements are resolved through discussion with a third-party inspector (XHH).

### 2.5 Statistical analysis

The extracted data were meta-analyzed to obtain standardized mean difference (SMD) and 95% confidence interval (CI). Chi-square test and I-square test were employed to evaluate the possible heterogeneity among the included studies, with I^2^ > 50% and P < 0.05 representing a significant level of heterogeneity. A fixed-effect model was used when the overall SMD revealed no obvious heterogeneity; otherwise, the random-effect model was used. Publication bias among the included studies was evaluated using Egger’s and Begg’s test.

All statistical analysis was performed using Review Manager version 5.4.1. and Stata/MP 17.0.

## 3 Results

### 3.1 Descriptive summary of studies

**Figure 1:**
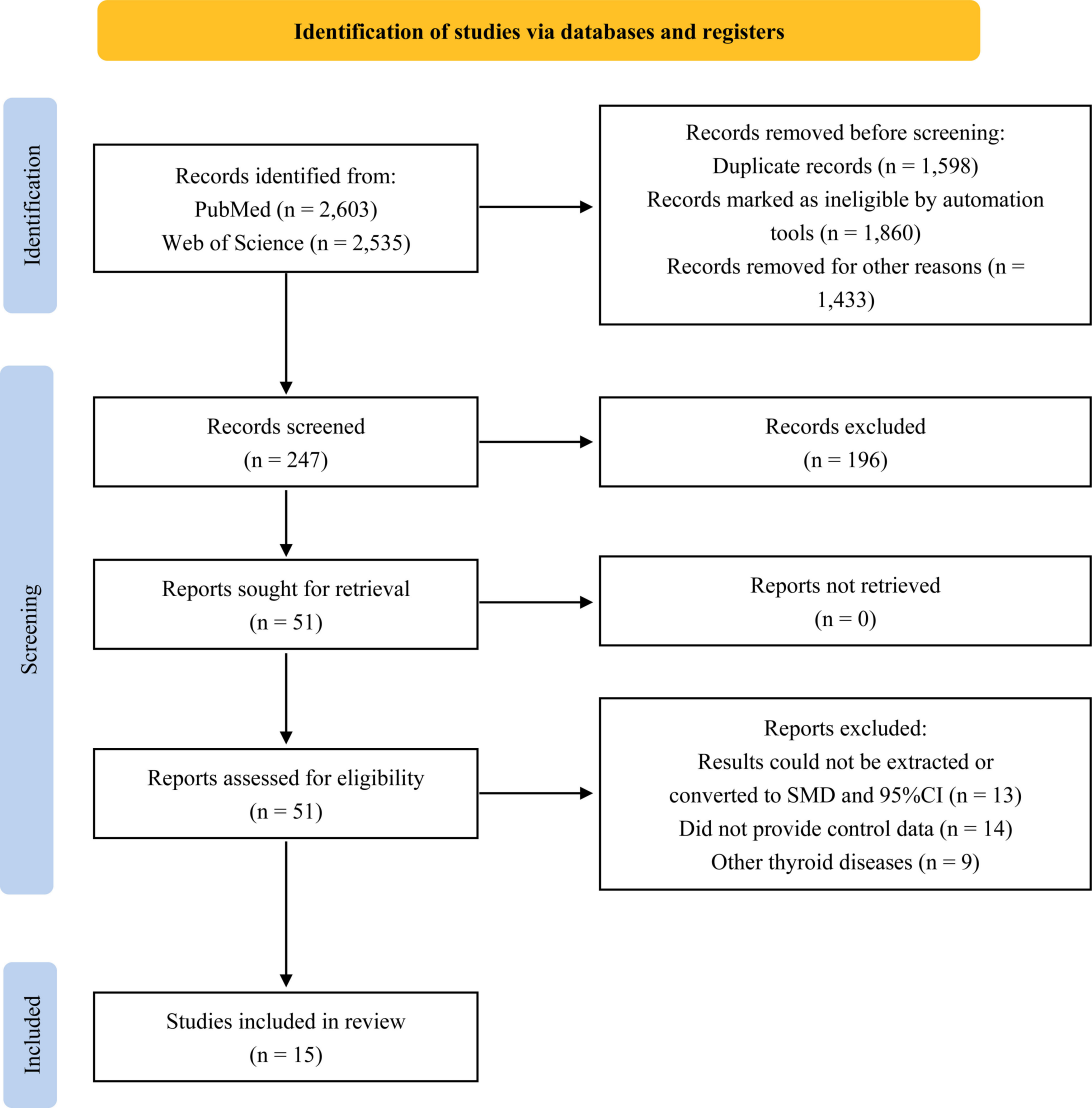
Flow diagram of study search and selection.

A total of 5,138 studies were retrieved through a literature search, and a preliminary review of titles and abstracts yielded 51 potentially eligible studies. Upon further review of the full text, 36 articles were excluded for the following reasons: not providing the level of EEDs exposure in thyroid cancer patients or healthy controls, inability to extract or convert results to SMD and 95%CI, or focusing on other thyroid diseases. As a result, 15 articles that met the inclusion criteria were eventually included in our analysis (13–16, 18–28). The flow diagram of the study selection process is presented in **Figure 1**.

The features included in the study are shown in **Table 1**. Among the 15 case-control articles, 13 focused solely on one type of EEDs, while the remaining 2 articles examined two EEDs each. There were 4 case-control studies on 18 PCBs homologues, 4 case-control studies on 8 PBDEs homologues, 2 case-control studies on 7 PAEs metabolites, 3 case-control studies on BPA, and 4 case-control studies on heavy metals. In terms of study location, 10 studies were conducted in China, 4 in the USA and 1 in Norway. The number of subjects in each study ranged from 118 to 1,484. NOS evaluation scores ranged from 7 to 9, with an average of 7.733. Most studies adjusted for confounding factors such as sex, age, BMI, and smoking status.

**Table 1:** Characteristics of the studies included in the meta-analysis.

Studies	Country	Study design	Study years	Source of study population	Source of sample	Method	Thyroid cancer			Control			NOS score
							N	Age	Female/Male	N	Age	Female/Male	
For PCBs													
** Lerro 2018** (14)	Norway	Nested Case-control Study	2008	Janus Serum bank cohort	Serum	GC-MS	108	41(18-65)	67/41	216	41(18-65)	134/82	8
** Deziel 2021** (23)	The USA	Case-control study	2010-2013	Connecticut Tumor Registry. Resident of Connecticut	Serum	GC-IDHRMS	250	21-84	250.00	250	21-84	250	8
** Han 2019** (19)	China	Case-control study	2016-2017	hospital	Serum	GC-HRMS	137	43.87 ± 12.27	/	186	45.84 ± 12.25	122/64	9
** Zhuo 2022** (27)	The USA	Nested Case-control Study	2000-2013	U.S. military cohort of service members	Serum	GC-ID/HRMS	742	26.25 ± 7.39	341/401	742	26.25 ± 7.34	341/401	8
For PBDEs													
** Deziel 2019** (13)	The USA	Case-control study	2010-2013	Connecticut Tumor Registry. Resident of Connecticut	Serum	GC-IDHRMS	250	21-84	250.00	250	21-84	250	8
** Zhang 2021** (24)	China	Case-control study	2019	hospital	Serum	GC-MS, ICP-OES, GFAAS	308	44.73 ± 12.72	239/69	308	46.10 ± 13.48	239/69	8
** Huang 2020** (20)	The USA	Nested Case-control Study	2000-2013	United States Army serum Bank	Serum	GC-IDHRMS	742	25(21,32)	341/401	742	25(21,32)	341/401	7
** Han 2019** (19)	China	Case-control study	2016-2017	hospital	Serum	GC-HRMS	137	43.87 ± 12.27	/	186	45.84 ± 12.25	122/64	9
For PAEs													
** Liu 2020** (15)	China	Case-control study	2016.3-2016.12	hospital	Urinary	HPLC-MS/MS	144	47.1 ± 11.6	104/40	144	44.9 ± 10.3	104/40	8
** Miao 2020** (21)	China	Case-control study	2017.6-2017.9	hospital	Urinary	UPLC-MS/MS	111	42.5 ± 11.4	86/25	111	42.5 ± 11.1	86/25	7
For BPA													
** Zhou 2017** (18)	China	Case-control study	2013.2-9	hospital	Urinary	HPLC-MS/MS	53	/	39/14	65	/	43/22	8
** Chen 2022** (28)	China	Case-control study	2016.3-2016.12	hospital	Urinary	HPLC-MS/MS	143	47.1 ± 11.6	103/40	224	/	161/63	8
** Zhang 2023** (26)	China	Case-control study	2017.6-2017.9	hospital	Urinary	UPLC-MS/MS	111	42.5 ± 11.4	86/25	111	42.5 ± 11.1	86/25	7
For Heavy Metals													
** He 2022** (25)	China	Case-control study	2017.3-2019.9	hospital	Urinary	ICP-OES, GFAAS	585	48.3(13.6)	372/213	585	48.8(14.3)	372/213	7
** Zhang 2019** (16)	China	Case-control study	2016.9-2018.3	hospital	Urinary	ICP-OES, GFAAS	262	47.1	177/85	262	46.8	177/85	8
** Zhang 2020** (22)	China	Case-control study	2017.3-2018.9	hospital	Urinary	AAS	218	46.18 ± 13.24	150/68	218	48.13 ± 14.28	150/68	7
** Zhang 2021** (24)	China	Case-control study	2019	hospital	Urinary	GC-MS, ICP-OES, GFAAS	308	44.73 ± 12.72	239/69	308	46.10 ± 13.48	239/69	8

PCBs, polychlorinated biphenyls; PBDEs, polybrominated diphenyl ethers; PAEs, phthalates; BPA, bisphenol A; GS-MS, gas chromatography-mass spectrometry; GC-IDHRMS, gas chromatography isotope dilution high resolution mass spectrometry; GC-HRMS, gas chromatography-high resolution mass spectrometry; GFAAS, graphite furnace atomic absorption spectrometry; ICP-OES, Inductively coupled plasma optical emission spectrometry; HPLC-MS/MS, high-performance liquid chromatography coupled with triple quadrupole tandem mass spectrometry; UPLC-MS/MS, ultra-performance liquid chromatography coupled with triple quadrupole tandem mass spectrometry; LC/FD/UV, liquid chromatography coupled with Ultraviolet and Fluorescence Detection; TDA-AAS, thermal decomposition amalgamation atomic absorption spectrometry.

The EEDs evaluated in this work are: polychlorinated biphenyls (PCBs), polybrominated diphenyl ethers (PBDEs), phthalates (PAEs), monobutyl phthalate (MBP), monoethylphthalate (MEP), monomethyl phthalate (MMP), mono-(2-ethyl-5-hydroxyhexyl) phthalate (MEHHP), mono-(2-ethyl-5-oxohexyl) phthalate (MEOHP), mono-(2-ethylhexyl) phthalate (MEHP), mono-(2-ethylpentyl-5-carboxy) phthalate (MECPP), bisphenol A (BPA), cadmium (Cd), lead (Pb), chromium (Cr), mercury (Hg), arsenic (As) and copper (Cu).

### 3.2 Environmental endocrine disruptors and thyroid cancer

#### 3.2.1 Polychlorinated biphenyls and thyroid cancer

**Figure 2:**
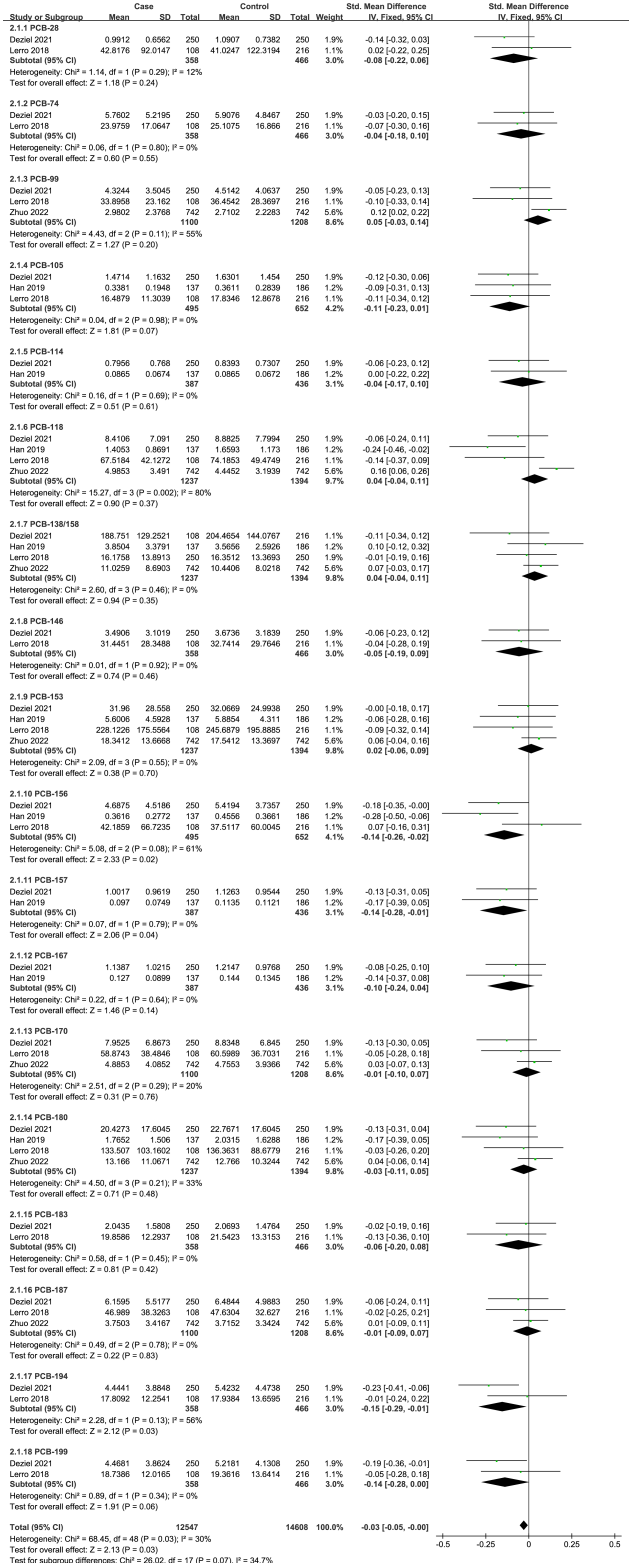
Forest plot of studies on PCBs levels for subjects with thyroid cancer patients versus healthy controls. The horizontal coordinate corresponding to the center of the green area is SMD, the horizontal lines represent the 95%CI, the green areas represent the weights, and the pooled SMD and 95%CI are represented as black diamonds.

Four case-control studies on PCBs were analyzed, including 18 PCBs (PCB-28, PCB-74, PCB-99, PCB-105, PCB-114, PCB-118, PCB-138/158, PCB-146, PCB-153, PCB-156, PCB-157, PCB-167, PCB-170, PCB-180, PCB-183, PCB-187, PCB-194, and PCB-199). The subgroup analysis was performed according to PCB congeners. Heterogeneity test result showed that there was no significant heterogeneity among the included studies (I^2^ = 30%, P = 0.03). Therefore, using a fixed effects model, the total SMD showed a negative association between PCBs and thyroid cancer risk {SMD = -0.03, 95%CI = (-0.05, -0.00), P = 0.03}. The forest plot is displayed in **Figure 2**. In the subgroup analysis, PCB-156 {SMD = -0.14, 95%CI = (-0.26, -0.02), P = 0.02}, PCB-157 {SMD = -0.14, 95%CI = (-0.28, -0.01), P = 0.04}, PCB-194 {SMD = -0.15, 95%CI = (-0.29, -0.01), P = 0.03}, and PCB-199 {SMD = -0.14, 95%CI = (-0.28, -0.00), P = 0.06} showed a negative association with thyroid cancer risk, while other PCB congeners were not statistically associated with thyroid cancer risk.

#### 3.2.2 Polybrominated diphenyl ethers and thyroid cancer

**Figure 3:**
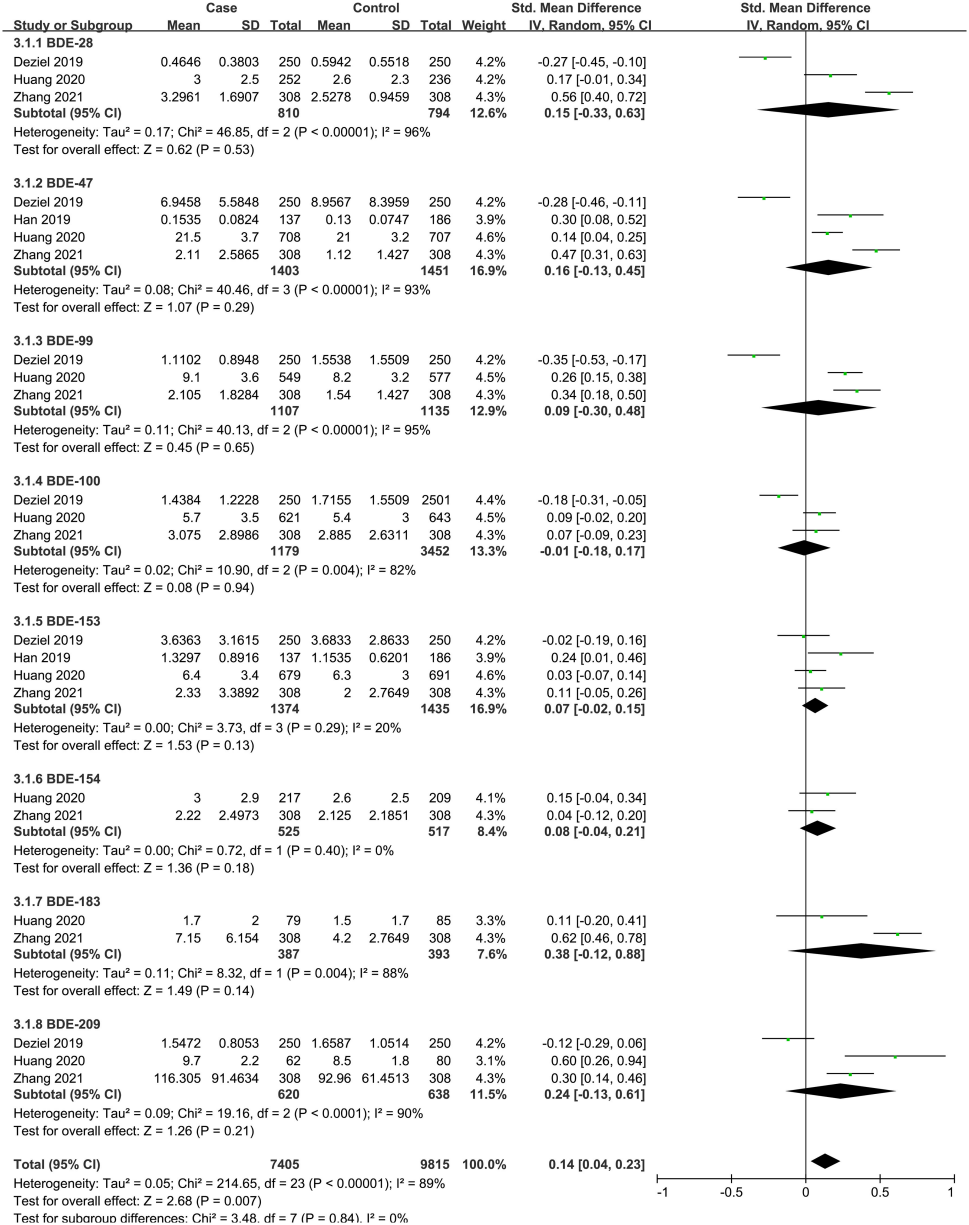
Forest plot of studies on PBDEs levels for subjects with thyroid cancer patients versus healthy controls. The horizontal coordinate corresponding to the center of the green area is SMD, the horizontal lines represent the 95%CI, the green areas represent the weights, and the pooled SMD and 95%CI are represented as black diamonds.

A total of four case-control studies were analyzed, including 8 PBDEs (BDE-28, BDE-47, BDE-99, BDE-100, BDE-153, BDE-154, BDE-183, BDE-209). The subgroup analysis was performed according to PBDE congeners. Heterogeneity test result showed that there was significant heterogeneity among the included studies (I^2^ = 89%, P = 0.007). Therefore, using a random effects model, the total SMD showed a positive association between PBDEs and thyroid cancer risk {SMD = 0.14, 95%CI = (0.04, 0.23), P = 0.007}. The forest plot is displayed in **Figure 3**. But in the subgroup analysis, no significant association was observed between the eight PBDE congeners and thyroid cancer risk.

#### 3.2.3 Phthalates and thyroid cancer

**Figure 4:**
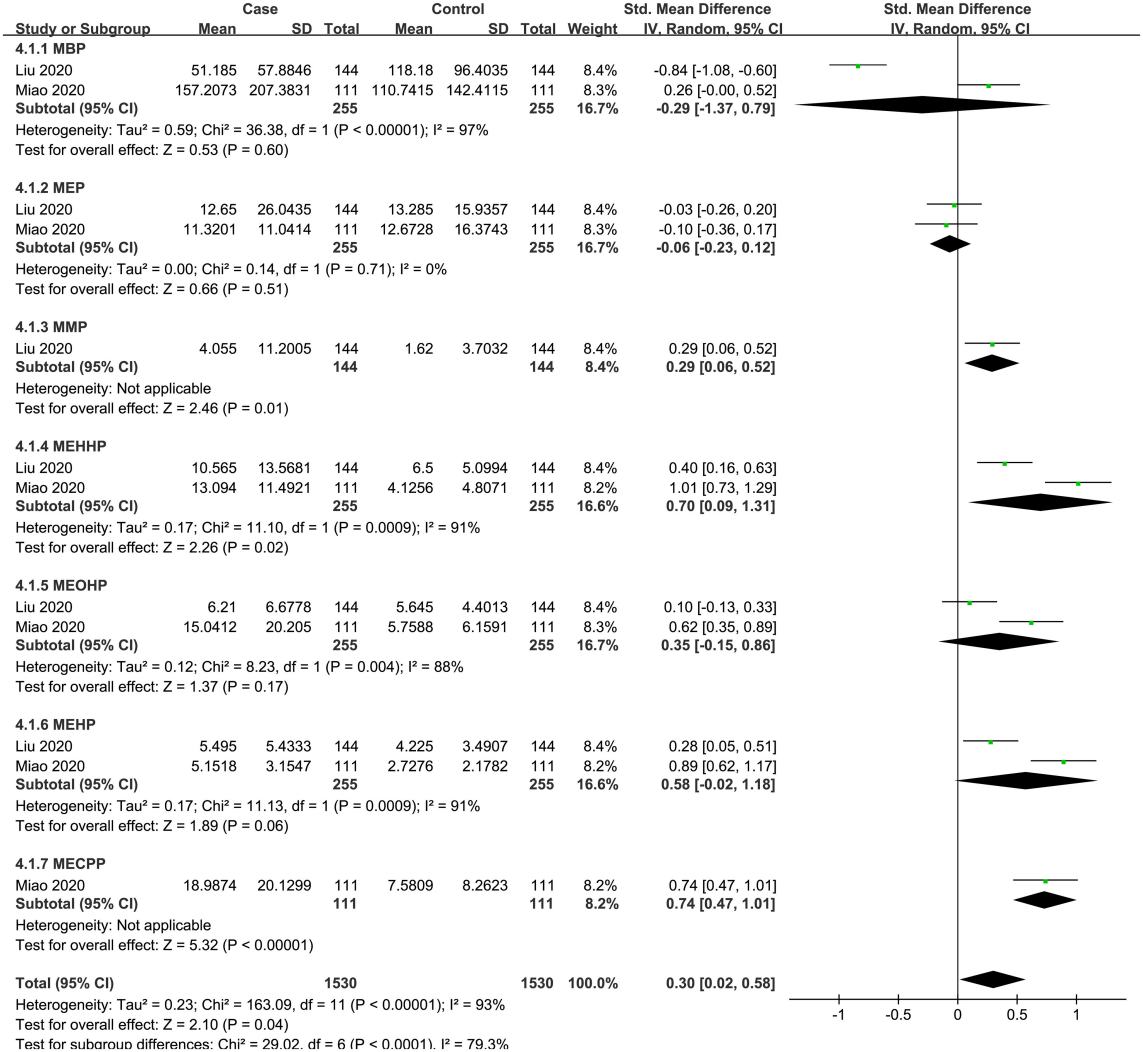
Forest plot of studies on PAEs levels for subjects with thyroid cancer patients versus healthy controls. The horizontal coordinate corresponding to the center of the green area is SMD, the horizontal lines represent the 95%CI, the green areas represent the weights, and the pooled SMD and 95%CI are represented as black diamonds.

A total of 2 case-control studies were included, including 7 PAEs (MBP, MMP, MEP, MEHHP, MEOHP, MEHP, MECPP). The subgroup analysis was performed according to PAE metabolites. Heterogeneity test result showed that there was significant heterogeneity among the included studies (I^2^ = 93%, P = 0.04). Therefore, using a random effects model, the total SMD showed a positive association between PAEs and thyroid cancer risk {SMD = 0.30, 95%CI = (0.02, 0.58), P = 0.04}. The forest plot is displayed in **Figure 4**. In the subgroup analysis, MEHHP {SMD = 0.54, 95%CI = (0.12, 0.97), P = 0.01} showed a positive association with thyroid cancer risk. MMP {SMD = 0.29, 95%CI = (0.06, 0.52), P = 0.01} and MECPP {SMD = 0.74, 95%CI = (0.47,1.01), P < 0.001} also showed a positive association with thyroid cancer, but each of them was based on only one article. Meanwhile, the SMD values of the other PAE metabolites showed no statistical significance.

#### 3.2.4 Bisphenol A and thyroid cancer

**Figure 5:**
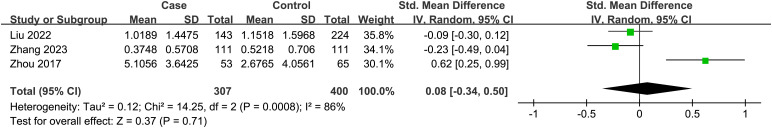
Forest plot of studies on BPA levels for subjects with thyroid cancer patients versus healthy controls. The horizontal coordinate corresponding to the center of the green area is SMD, the horizontal lines represent the 95%CI, the green areas represent the weights, and the pooled SMD and 95%CI are represented as black diamonds.

A total of 3 studies were included, and the random-effect model was used according to heterogeneity (I^2^ = 86%, P = 0.71). The result showed that there was no statistically significant association between thyroid cancer and BPA {SMD = 0.08, 95%CI = (-0.34, 0.50), P < 0.001}. The forest plot is displayed in **Figure 5**.

#### 3.2.5 Heavy metals and thyroid cancer

**Figure 6:**
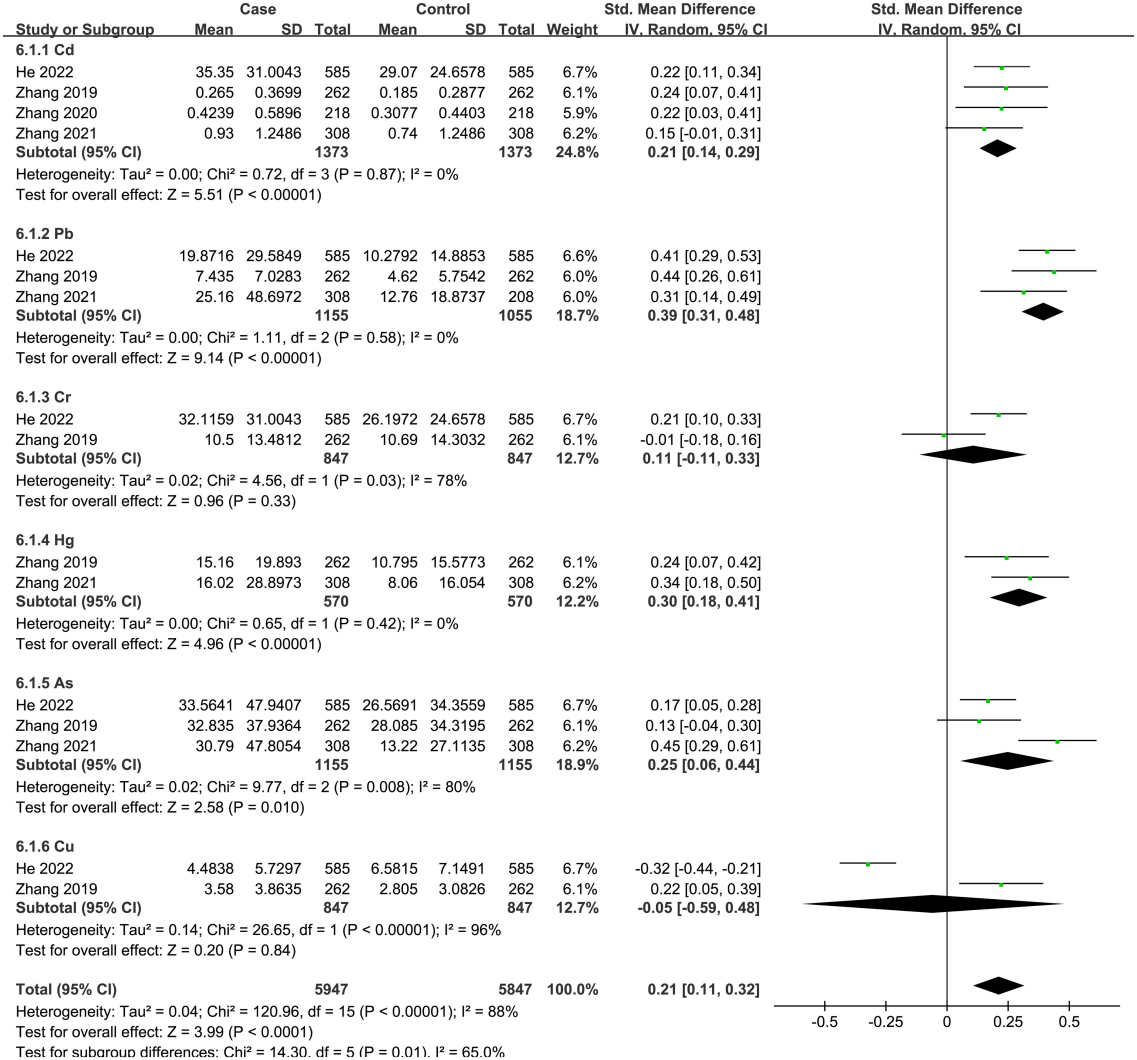
Forest plot of studies on heavy metals levels for subjects with thyroid cancer patients versus healthy controls. The horizontal coordinate corresponding to the center of the green area is SMD, the horizontal lines represent the 95%CI, the green areas represent the weights, and the pooled SMD and 95%CI are represented as black diamonds.

A total of 6 articles were analyzed, including 6 heavy metals (Cd, Pb, As, Hg, Cu, Cr). The heterogeneity test showed significant heterogeneity among the included studies (I^2^ = 88%, P < 0.001). Therefore, a random effect model was used, and the total SMD indicated a positive correlation between heavy metals and thyroid cancer risk {SMD = 0.21, 95%CI = (0.11, 0.32), P < 0.001}. The forest plot is displayed in **Figure 6**. The results showed that Cd {SMD = 0.21, 95%CI = (0.14, 0.29), P < 0.001}, Pb {SMD = 0.39, 95%CI = (0.31, 0.48), P < 0.001), Hg {SMD = 0.30, 95%CI = (0.18, 0.41), P < 0.001}, and As {SMD = 0.25, 95%CI = (0.06, 0.44), P = 0.01) were positively associated with the risk of thyroid cancer, while Cr and Cu indicated no statistical significance.

### 3.3 Publication bias

Publication bias was measured using Egger’s test and Begg’s test. There was no evidence of publication bias found for PBDEs (Egger test P = 0.658, Begg test P = 0.535), PAEs (Egger test P = 0.063, Begg test P = 0.007), BPA (Egger test P = 1.000, Begg test P = 0.416), and heavy metals (Egger test P = 0.335, Begg test P = 0.300). However, the analysis revealed a significant publication bias in relation to PCBs (Egger test P = 0.000, Begg test P = 0.001), as presented in **Table 2**.

**Table 2:** Endocrine disruptors and thyroid cancer risk: Summary of SMD and publication bias results.

EEDs	No. of studies	SMD (95% CI)	Heterogeneity		Publication bias	
			P value	I^2^ value, %	Egger P value	Begg P value
PCBs	4	-0.03 (-0.05, -0.00)	0.03	30	0.000	0.001
PBDEs	4	0.14 (0.04, 0.23)	0.007	89	0.658	0.535
PAEs	2	0.30 (0.02, 0.58)	0.04	93	0.063	0.007
BPA	3	0.08 (-0.34, 0.50)	0.71	86	1.000	0.416
Heavy metals	4	0.21 (0.11, 0.32)	0.000	88	0.335	0.300

EEDs, environmental endocrine disruptors; SMD, standardized mean difference; PCBs, polychlorinated biphenyls; PBDEs, polybrominated diphenyl ethers; PAEs, phthalates; BPA, bisphenol A.

## 4 Discussion

PCBs have been identified as substances that interfere with thyroid hormone, by competitively binding to thyroid transporters and disrupting thyroid hormone signal transduction. This can lead to reduced thyroid hormone circulation, abnormal thyroid proliferation, and even tumor occurrence (4, 29–31). PCBs are internationally recognized as carcinogens (32). However, a small cohort study conducted in New York State (n = 27 cases), which assessed the risk of thyroid cancer among fishers based on local fish consumption and type, found no association between PCBs exposure and thyroid cancer (33). According to a joint cohort study conducted on PCB-exposed capacitor manufacturing workers at plants in Indiana, Massachusetts and New York, there was no increase in thyroid cancer mortality or association with PCBs (34). The results of this meta-analysis reveal a negative association between PCBs and thyroid cancer, particularly with PCB-156, PCB-157, PCB-194 and PCB-199. Among the four articles included, Deziel et al. (23) did not find any evidence supporting a link between PCBs and thyroid cancer. Lerro et al. (14) found a positive association between PCBs and thyroid cancer, but only in the youngest birth cohort (1943–1957). However, Zhuo et al. (27) found a significant association between PCB-118, PCB-74, and PCB-99 with the risk of thyroid cancer, with PCB-118 being especially notable. Han et al. (19) identified a positive association between PCB-114 and thyroid cancer. These findings contradict our analysis, and there is currently insufficient evidence to establish a clear association between PCBs exposure and thyroid cancer. The significant publication bias detected through Egger’s test and Begg’s test may be attributed to the negative correlation between the results of the included articles and thyroid cancer. Consequently, the relationship between PCBs exposure and thyroid cancer risk remains an important unresolved question.

PBDEs are a type of common brominated flame retardants, and their toxic effects mainly include thyroid hormone disturbance and neurotoxicity. There is no consensus on their carcinogenicity (35, 36). Thyroid follicular cell hyperplasia was observed in B6c3f1 mice after repeated dietary exposure to decabromodiphenyl ether(BDE-209) (37). Studies have shown that BDE-71 and BDE-79, acting as a mixed inducer of liver enzyme activity, can reduce the circulating concentration of T4 by increasing hepatic glucosaldehyde acidification, leading to hypothyroidism (38). PBDEs can also competitively bind to serum transporters and reduce their activity, thereby affecting thyroid hormone levels in circulation (39). Given the critical role of thyroid hormone in regulating multiple biological functions, this may link PBDE to adverse outcomes, including cancer (39–41). The results of this meta-analysis showed a positive association between PBDEs and thyroid cancer risk, but no significant association between PBDE homologues and thyroid cancer risk was observed in subgroup analysis. The association between PBDEs and thyroid cancer was not supported only by Deziel et al. (13). Therefore, further research is needed to investigate this association. It is important to note that the high heterogeneity observed in the study may be attributed to variations in the race, gender, and age of the study population.

PAEs are commonly used as plasticizers and softeners in various consumer products (42). PAEs and their metabolites can affect thyroid hormone homeostasis at multiple levels (43, 44). Exposure to DEHP and its metabolites resulted in decreased levels of sodium-iodine homologous transporter (NIS) and thyroxine transporter (TTR), increased levels of deiodinase I and glucuronyl transferase (UGT) in the liver, and affected circulating thyroid hormone levels (45). DEHP also increase the number of thyroid follicular epithelial cells (43). In a study investigating the toxicological effects of subchronic to low- and high-dose DEHP exposure in rats from weaning to infancy, DEHP induced thyroid hyperplasia at a dose of 30mg/kg/day; and transcriptome data showed that several cancer-related genes were altered (46). This meta-analysis showed that PAEs exposure was positively associated with thyroid cancer risk, with MMP, MEHHP, and MECPP significantly increasing the risk of thyroid cancer. The high heterogeneity may be attributed to factors such as the small number of included articles, the short half-life of PAEs, the larger proportion of women, and the inability to fully control for confounding factors. More research is necessary to explore the association between PAEs and thyroid cancer.

BPA is widely used in the manufacture of plastic bottles, food packaging, toys, etc. As one of the common EEDs, BPA can affect thyroid function in various ways (8, 47). BPA can up-regulate the expression of genes related to thyroid hormone biosynthesis and specific transcription factors that control thyroid development and thyroid follicular cell differentiation, such as Pax8, Nkx2-1 and Foxe1 (48). A study of early BPA exposure showed a significant correlation between blood BPA levels and the expression of BPA-reactive proteins in thyroid tissue, such as ANXA6 and VCP, through which BPA may accelerate cancer progression (49). BPA also mediates estradiol-like effects by binding to nuclear estrogen receptors (ERα and ERβ) and activates AKT/mTOR pathway by binding to estrogen membrane receptors (mERα and GPR30). These ultimately alter gene expression to stimulate the proliferation of thyroid cancer cells (50). Chen et al. (28) observed that BPA was associated with increased oxidative stress, as indicated by elevated urinary concentrations of 8-OHdG, 8-isoPGF_2α_ and HNE-MA. Further analysis suggested that urinary 8-isoPGF_2α_ mediated part of the positive associations between BPA exposure and the risk of thyroid cancer. However, this meta-analysis did not show a statistically significant association between BPA and thyroid cancer. On the other hand, Zhou et al. (18) reported a significant association between BPA and thyroid cancer. In the other two articles, urinary BPA concentrations were lower in thyroid cancer patients compared to controls. These discrepancies may be attributed to differences in the study population, the shorter biological half-life of urinary BPA, and confounding factors that were not fully controlled.

Heavy metals, as a type of endocrine disruptors, have carcinogenicity and bioaccumulation. The International Agency for Research on Cancer (IARC) has listed arsenic (As) and cadmium (Cd) as common carcinogens, and mercury (Hg) and lead (Pb) as possible carcinogens (24, 51). Long-term exposure to heavy metals can lead to various adverse effects on the body and increase the risk of malignant tumors (52, 53). The incidence of thyroid cancer is much higher on volcanic islands that are contaminated by heavy metals (54). Some heavy metals (Cu, Hg, Pd, W and Zn) have been found to stimulate the proliferation of thyroid stem/progenitor cells, but have no effect on mature thyroid cells (55). Heavy metals have been found to play a carcinogenic role by inducing oxidative stress, affecting cell apoptosis, causing DNA damage, and altering gene expression (56). The results of our meta-analysis showed that Cd, Pb, As and Hg were positively associated with the risk of thyroid cancer, while Cr and Cu were not statistically correlated. The divergent results reported in the two articles on Cr and Cu may be due to differences in the study population.

Perfluoroalkyl substances (PFAS) are also important thyroid disruptors that competitively bind thyroxine binding globulin (TGB) and transthyretin (TTR), thereby regulating iodothyronine deiodinase activity. This regulation can potentially impact serum levels of free triiodothyronine (FT3), free thyroxine (FT4), and thyroid stimulating hormone (TSH), ultimately affecting thyroid homeostasis (57–59). While numerous studies have investigated the effects of PFAS on thyroid function, there is limited research available on its association with thyroid cancer (58). After conducting a thorough search, only one case-control study (60) that met our inclusion criteria was identified, and thus, it was not included in our meta-analysis. In this article, they found that exposure to PFASs was inversely associated with the risk of thyroid cancer. However, further research is needed to explore the potential relationship between PFAS and thyroid cancer.

This meta-analysis included numerous high-quality studies and reported the potential association between five types of EEDs and thyroid cancer risk, with subgroup analyses conducted and no publication bias observed except for studies on PCBs. However, there are some limitations. First, the results showed that there is a high degree of heterogeneity in the studies on PBDEs, PAEs, BPA and heavy metals, which may be due to the fact that most concentration data were obtained from serum or urine, and the concentrations of EEDs in the subjects at the measured nodes varied; It is also possible that some individual factors, such as family history of thyroid disease, financial status, etc. could have contributed to the differences in results. Second, a total of 15 articles were included in this meta-analysis, but the small number of studies involving each type of EEDs (some only 2-3 articles) weakens the quality of the results and makes them unstable. The number of included studies was limited, preventing us from conducting additional analyses to explore possible sources of heterogeneity among the included studies. It is important to note that heterogeneity of studies may be unavoidable. Additionally, our study focused only on the relationship between each individual EED and thyroid cancer risk. However, humans are typically exposed to a complex mixture of pollutants, and we did not discuss the effects of interactions between multiple EEDs on thyroid cancer. It is crucial to acknowledge that retrospective studies cannot establish causality, and we can only infer a possible association between EEDs and thyroid cancer from our analyses. Further studies are necessary to investigate the specific biological mechanism and effects of EEDs exposure on thyroid cancer. Despite these limitations, our meta-analysis provides valuable insights for future research, particularly regarding the potential combined effects of multiple EEDs on thyroid cancer.

## 5 Conclusion

In conclusion, the results of our current meta-analysis suggest that PBDEs, PAEs, and heavy metals exposure have a significant impact on thyroid cancer risk. While the analysis method reduced heterogeneity to some extent, it remained high in most cases in this study. Further study is required to investigate the relationship between different EEDs and thyroid cancer.

## Data availability statement

The original contributions presented in the study are included in the article/supplementary material. Further inquiries can be directed to the corresponding author.

## Author contributions

YY: Writing – original draft, Writing – review & editing. XB: Writing – original draft, Writing – review & editing. JL: Writing – review & editing. RZ: Writing – review & editing. RD: Writing – review & editing. XH: Writing – review & editing.
